# Thermal Chemisorption and Reduction of Carbon Dioxide on UiO-66(Zr) and MIL-100(Fe)

**DOI:** 10.3390/nano15070479

**Published:** 2025-03-22

**Authors:** Smita Takawane, Masatoshi Miyamoto, Atsushi Kondo, Koki Urita, Tomonori Ohba

**Affiliations:** 1 Graduate School of Science, Chiba University, Yayoi, Inage, Chiba 263-8522, Japan; 22wd2302@student.gs.chiba-u.jp (S.T.); 20s3034m@student.gs.chiba-u.jp (M.M.); 2 Department of Science and Technology, Faculty of Science and Technology, Oita University, Oita 870-1192, Japan; kondoa@oita-u.ac.jp; 3 Graduate School of Integrated Science and Technology, Nagasaki University, 1-14 Bunkyo, Nagasaki 852-8521, Japan; urita@nagasaki-u.ac.jp

**Keywords:** thermo-catalysts, CO_2_ reduction, CO_2_ adsorption, metal–organic frameworks, MIL-100(Fe), UiO-66(Zr)

## Abstract

The continuous increase in global energy consumption has caused a considerable increase in CO_2_ emissions and environmental problems. To address these challenges, adsorbents and catalytic materials that can effectively reduce the CO_2_ levels in the atmosphere should be developed. Metal–organic frameworks (MOFs) have emerged as promising materials for CO_2_ capture owing to their high surface areas and tunable structures. Herein, the CO_2_ adsorption properties of MIL-100(Fe) and UiO-66(Zr) were investigated. Both MOFs exhibited excellent thermal stability and high CO_2_ adsorption capacities at 300 K, and they maintained good adsorption properties at 500 K compared to those of activated carbon fiber owing to their high adsorption potentials. A slight change in the UiO-66(Zr) structure and no change in the MIL-100(Fe) structure were observed under the CO_2_ atmosphere at 500 K. At that time, CO emissions and changes in the carboxyl and OCO functional groups were observed on MIL-100(Fe), suggesting a mechanism of CO_2_ reduction to CO on the bare Fe(II) sites. These findings confirm the potential of MOFs for the thermo-catalytic reduction of CO_2_ to achieve effective CO_2_ capture and conversion.

## 1. Introduction

Energy consumption has increased in recent decades to satisfy the increasing industrial requirements and daily needs caused by the rapid population growth. Fossil fuels are still one of the primary energy sources. As a result, the CO_2_ concentration in the atmosphere increased substantially from 340 ppm in 1980 to 408 ppm in 2019, leading to several negative environmental and global economic impacts [1]. For example, the total production of rice, maize, and wheat grains exhibited a 50–100% decrease due to global warming. In most climate models, global warming increases economic inequality because it affects developing countries more than developed countries [2]. Thus, reducing CO_2_ emissions and its atmospheric concentration is vital to mitigate these global problems.

Numerous extensive studies on CO_2_ capture and storage employing several techniques have been reported: absorption using aqueous amine solutions, adsorption using porous materials, and separation using polymeric membranes. However, there are still several challenges that should be overcome. For example, although the absorption techniques exhibit extremely high absorption performance, they require a substantially high amount of energy for regeneration [3,4]. Moreover, the pressure- and temperature-swing adsorption and membrane separation techniques exhibit relatively low CO_2_ selectivity and capacity. Nevertheless, CO_2_ capture and separation using porous materials, such as zeolites, activated carbons, carbon molecular sieves, porous alumina, carbon nanotubes, and silica gels, exhibit low energy consumption [5,6,7,8,9]. Metal–organic frameworks (MOFs) have high CO_2_ separation capacity and selectivity because of their uniform pore structures with large surface areas [1,10,11]. MOFs can have various designs while maintaining high chemical stability by choosing appropriate metals and ligands and applying various chemical modifications [12]. Extensive efforts have been dedicated to studying the adsorption of CO_2_ by several MOFs. Flexible MOFs have dynamic frameworks that can change their structures in response to external stimuli, such as temperature, pressure, and guest molecules, leading to high CO_2_ adsorption [13]. Mg-MOF-74 shows that CO_2_ selectivity against its N_2_ selectivity can be increased when water molecules are present in the system owing to water adsorption on the coordinatively unsaturated metal sites; however, this strategy decreases the CO_2_ adsorption capacity [14]. Moreover, MOF membranes exhibit long-term stability, high capacity, selectivity, and chemical resistance to acids, bases, and aqueous solutions. MOFs can be used in coupling reactions of CO_2_ and epoxides to form binary catalysts, such as propylene carbonate and butylene carbonate [15], and in optical media [16].

This study uses two MOFs, i.e., MIL-100(Fe) and UiO-66(Zr), which are representative MOFs with high porosity and thermal stability, as CO_2_ thermal-reducing catalysts. MIL-100(Fe) is a promising candidate for various applications in decontamination, drug delivery, and catalysis because of its superior capturing ability due to its mesoporous structure, which provides it with a large surface area and allows its post-synthetic modification [17,18,19]. Furthermore, UiO-66(Zr), in which zirconium-oxo clusters form the micropores with dicarboxylate ligands, exhibits excellent CO_2_ adsorption properties compared to its CH_4_ and N_2_ adsorption properties, which can be attributed to its polarity and small functional groups [20]. The modification and conjugation of the MOFs improved their CO_2_ adsorption capacity. Compositing UiO-66(Zr)-NH_2_ with GO increased its CO_2_ adsorption capacity by 50% [21]. Thus, we investigated the CO_2_ adsorption mechanism to clarify the physical adsorption and chemisorption mechanisms on MIL-100(Fe) and UiO-66(Zr) and the possibility of CO_2_ reduction on those representative MOFs at 300–500 K.

## 2. Materials and Methods

MIL-100(Fe) was prepared based on a method reported in the literature [22]. Briefly, a NaOH aqueous solution (1 M) was prepared by dissolving NaOH (23.7 g) in water, and 1,3,5-benzene tricarboxylic acid (7.6 mmol) was added dropwise to an aqueous solution of FeCl_2_·4H_2_O (11.4 mmol), which was prepared by dissolving FeCl_2_·4H_2_O (97.2 g) in water. After stirring for 24 h at ambient temperature, the mixture solution was centrifuged at 4000 rpm. The brown sediment was collected and washed several times with water and ethanol. UiO-66(Zr) was prepared according to procedures reported in the literature [23]. A HCl aqueous solution (10 M) and ZrCl_4_ (5.0 mmol) were added to dimethylformamide (100 mL) and stirred for 5 min at room temperature. 1,4-benzenedicarboxylic acid (7.5 mmol) was then added to the mixture under stirring for 15 min, and the final mixture was heated at 353 K for 24 h. The reaction mixture was finally cooled to room temperature, and fine white powder was collected by filtration, followed by washing with water and ethanol.

MIL-100(Fe) and UiO-66(Zr) powders were characterized using powder X-ray diffraction (XRD, SmartLab, Rigaku Co., Tokyo, Japan) with Cu Kα radiation (λ = 0.1541 nm) at 40 kV and 40 mA. Their microstructures were observed directly using high-resolution transmission electron microscopy (HR-TEM, ARM200CF, JEOL Ltd., Tokyo, Japan). The acceleration voltage was set to 120 kV to minimize electron irradiation damage and obtain a clear microstructural image. The thermal stabilities of these MOFs were evaluated using thermogravimetric (TG) analysis (ThermoEVO2, Rigaku Co., Tokyo, Japan), in which each MOF was placed in an alumina pan and heated to 900 K at a heating rate of 10 K min^−1^ under an O_2_ atmosphere (flow rate = 100 mL min^−1^), and the alumina powder was used as a reference. The pore structures and specific surface areas were evaluated based on N_2_ adsorption isotherms at 77 K using the BELSORP MAX apparatus (MicrotracBEL Co., Osaka, Japan). The samples were preheated at 500 K at a pressure lower than 10^−3^ Pa in an N_2_ atmosphere (gas purity > 99.995%) to remove guest molecules. The pore size distributions were determined based on the desorption branch of the N_2_ adsorption isotherms using the grand canonical Monte Carlo simulation models in the BELMaster 7 software (MicrotracBEL Co., Osaka, Japan). The adsorbed CO_2_ amounts at 300, 400, and 500 K were determined using BELSORP MAX, as mentioned in the N_2_ adsorption measurements. CO_2_ gas (purity > 99.9%) was used in the experiments. Activated carbon fiber (A5, AD’ALL Co., Kyoto, Japan) was used as a reference sample for determining adsorbed CO_2_ amounts. The adsorption–chemisorption dynamics of CO_2_ were evaluated using TG analysis. For this analysis, the samples were preheated at 500 K under an Ar atmosphere (flow rate of CO_2_ and Ar gases = 100 mL min^−1^) for eight hours, and the TG analysis was then conducted at 500 K for four hours. Moreover, CO emissions were evaluated using mass spectroscopy during the TG measurement (BEL-MASS II, MicrotracBEL Co., Osaka, Japan) combined with TG analysis. The CO_2_-CO adsorbed structures on MOFs were evaluated using Fourier-transform infrared (IR) spectroscopy (FT/IR-4000, IRT-5000 JASCO Co., Tokyo, Japan).

## 3. Results and Discussion

**Figure 1:**
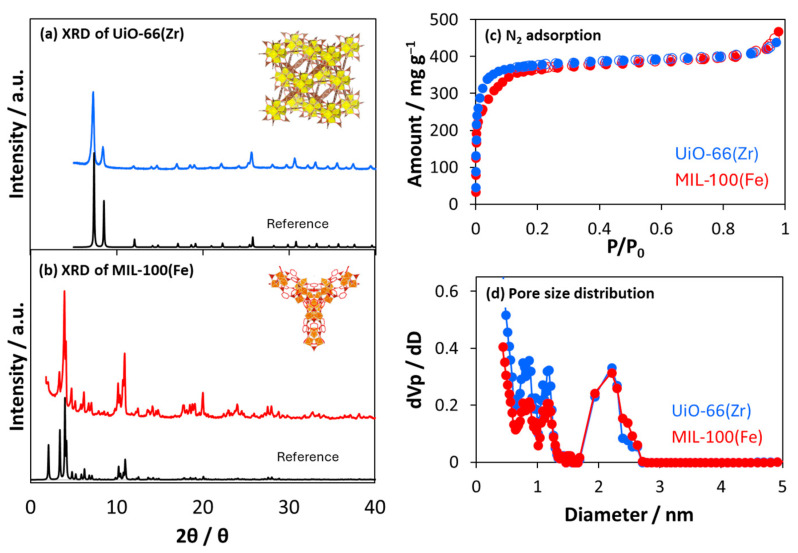
XRD patterns of (**a**) UiO-66(Zr) and (**b**) MIL-100(Fe): the blue, red, and black curves represent the prepared UiO-66(Zr), the prepared MIL-100(Fe), and the references. (**c**) N_2_ adsorption isotherms on UiO-66(Zr) and MIL-100(Fe) at 77 K. The closed and open symbols represent adsorption and desorption branches, respectively. (**d**) Pore size distributions were obtained based on the N_2_ adsorption isotherms at 77 K.

**Figure 2:**
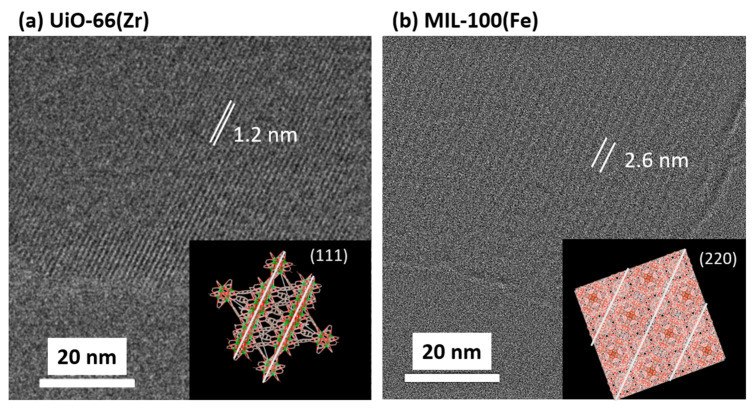
HR-TEM images of UiO-66(Zr) (**a**) and MIL-100(Fe) (**b**) and their structure images. The insets represent the unit cells of the MOFs. Green, yellow, brown, and red spheres depict Zr, Fe, C, and O, respectively.

Figure 1a,b show the prepared UiO-66(Zr) and MIL-100(Fe) XRD patterns, respectively. The peaks corresponded to the reported UiO-66(Zr) [24] and MIL-100(Fe) crystals [25,26]. The HR-TEM images in Figure 2 indicated that the interference fringes were observed with distances of 1.0 ± 0.2 nm and 2.4 ± 0.2 nm for UiO-66(Zr) and MIl-100(Fe), respectively. The uniform lattice fringes confirm that the prepared MOF materials are highly crystalline. Those distances were assigned by the (111) lattice plane on UiO-66(Zr) at 7.1 (1.2 nm distance) and the (220) lattice plane on MIL-100(Fe) at 3.4 (2.6 nm distance) in the XRD patterns. UiO-66(Zr) contains octahedral and tetrahedral cages (size = 1.2 and 0.6 nm, respectively) [27], whereas MIL-100(Fe) contains two types of mesoporous cages (size = 2.5 and 2.9 nm) [28]. The N_2_ adsorption isotherms on UiO-66(Zr) and MIL-100(Fe) at 77 K are shown in Figure 1c, which indicates that both isotherms are typical Langmuir isotherms and are in good agreement with each other, indicating the similar micropore volumes. However, the adsorption jump on UiO-66(Zr) was steeper than on MIL-100(Fe). The steep adsorption jumps at low pressure indicated the presence of narrower micropores, leading to micropore filling. Moreover, the adsorption and desorption isotherms completely matched, indicating few mesopores from the MIL-100(Fe) crystal structure. UiO-66(Zr) and MIL-100(Fe) pore size distributions were similar despite the different pore sizes determined based on their crystal structures. The discrepancy between the crystal and pore structures is due to the strong adsorption potential in the MIL-100(Fe) nanopores. The structural parameters are shown in Table 1. The specific surface area of UiO-66(Zr) was slightly larger than that of MIL-100(Fe), calculated using the Brunauer–Emmett–Teller (BET) equation [29]. In contrast, the pore volumes of the two materials, which were estimated based on the amounts at P/P_0_ = 0.8, were close. The average pore diameter of UiO-66(Zr) was smaller than that of MIL-100(Fe), which agreed with the estimation based on the crystal structures. However, the pore volume of UiO-66(Zr) 0.50 cm^3^ g^−1^ estimated from the N_2_ adsorption was larger than the value of 0.42 cm^3^ g^−1^, which was estimated from the crystal structure, implying the presence of defects and/or interparticle pores.

**Figure 3:**
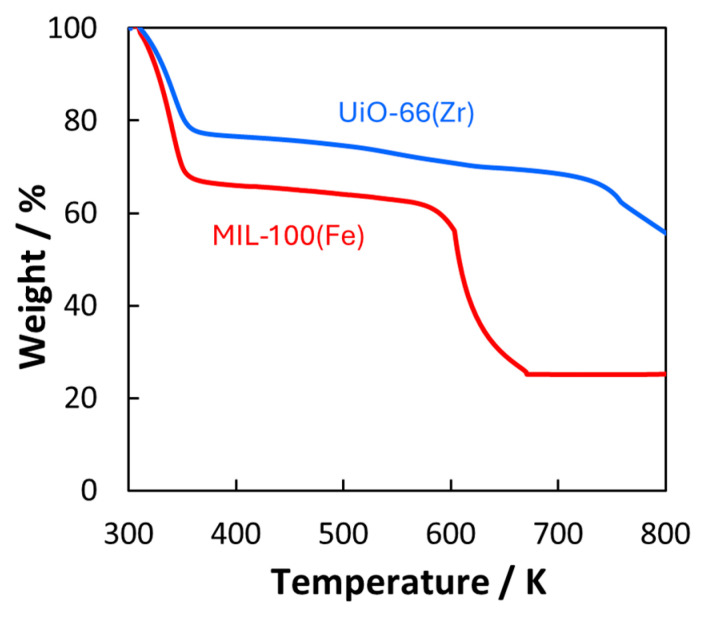
Thermal decomposition of MOFs. UiO-66(Zr) and MIL-100(Fe) are depicted in blue and red curves.

**Figure 4:**
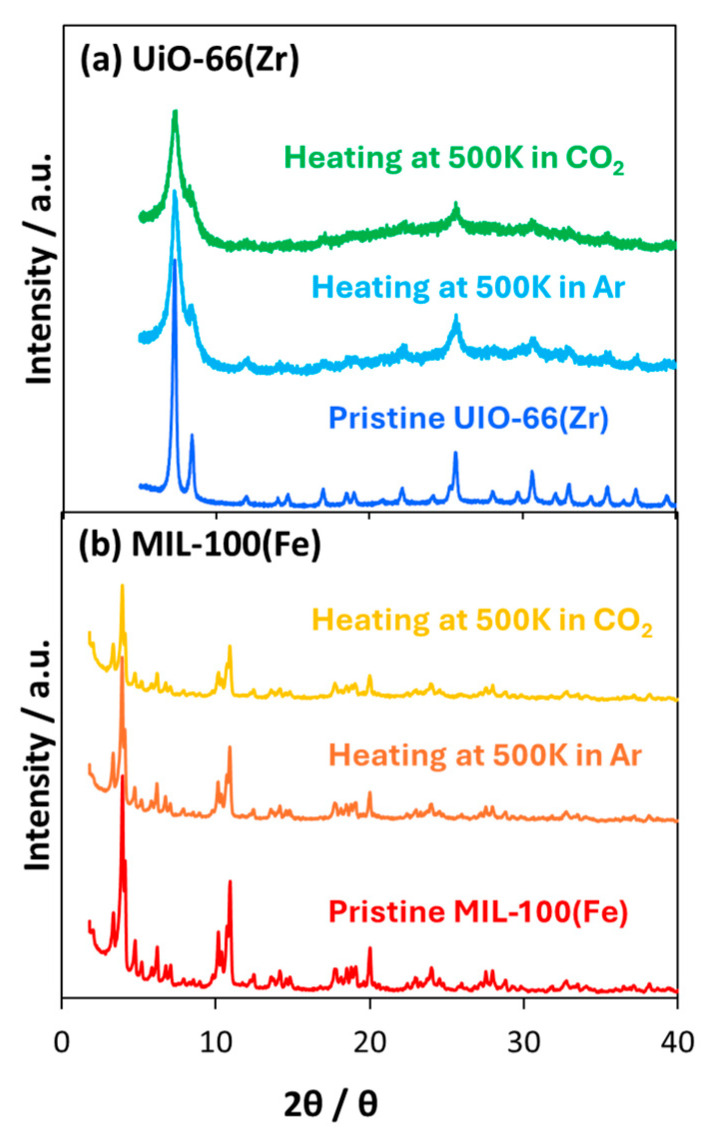
XRD patterns of (**a**) UiO-66(Zr) and (**b**) MIL-100(Fe) after heating at 500 K under CO_2_ and Ar atmospheres.

The thermal stability of the MOFs was assessed using thermal decomposition analysis under an oxygen atmosphere at 300–800 K (Figure 3). The weight decreases at temperatures less than 400 K, which can be attributed to the removal of guest molecules. At 400–550 K, the MOFs were stable. Finally, the MOFs started to decompose at temperatures higher than 550 K. The thermal stability of the MOFs at 500 K was evaluated based on their crystallinity (Figure 4). Figure 4 shows the XRD patterns before and after heating for 4 and 8 h under CO_2_ and Ar atmospheres, respectively. The peak positions of UiO-66(Zr) did not change, indicating that the UiO-66(Zr) crystal structure was generally maintained throughout the heating process. At the same time, the peaks were broadened, suggesting that the crystallinity was slightly decreased compared to that in the pristine one. The MIL-100(Fe) crystal structure was also hardly changed by heating. This indicated that the structure degradation probably caused by pulverization occurred on the UiO-66(Zr) compared with MIL-100(Fe) despite the higher stability in Figure 3. However, the crystalline structures of both MOFs were maintained because of unchanged XRD peak positions. When heating for longer than 10 h, the MOF structures were unchanged. Thus, we concluded that UiO-66(Zr) and MIL-100(Fe) were thermally stable at temperatures less than 550 K.

**Figure 5:**
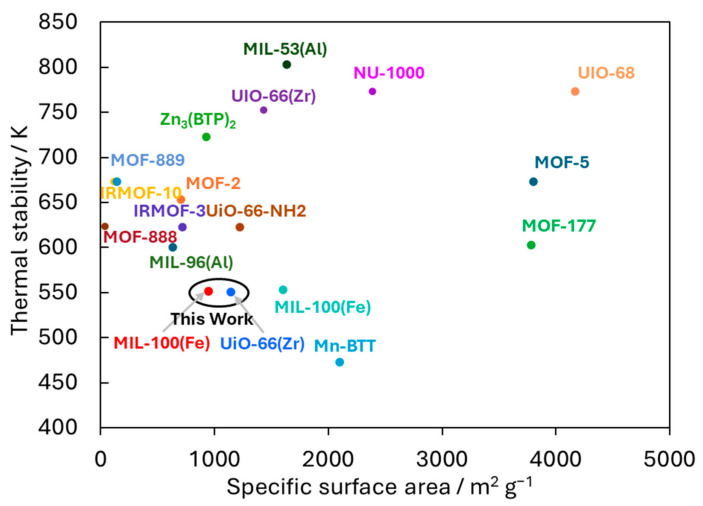
Relationship between the thermal stabilities and specific surface areas of various MOFs. The UiO-66(Zr) and MIL-100(Fe) prepared in this work are circled in black.

The relationship between the thermal stability and specific surface areas of various MOFs is illustrated in Figure 5 [17,30,31,32,33,34,35,36,37,38,39,40,41]. Some MOFs exhibit high thermal stability and specific surface areas. The previously reported UiO-66(Zr) and MIL-100(Fe) also showed higher stability and specific surface areas compared to the prepared ones, which is likely due to variations in the synthesis techniques, solvents used, and distinct analytical methods employed for characterization [17,36]. The thermal stabilities of UiO-66(Zr) and MIL-100(Fe) were confirmed until 750 K and 550 K, respectively, and their specific surface areas were both 1500 ± 100 m^2^ g^−1^. Although the prepared UiO-66(Zr) and MIL-100(Fe) do not show the best thermal stability and specific surface areas among MOFs, they are common materials that exhibit relatively high thermal stability, specific surface areas, and numerous nanopores. Therefore, this study used the two MOFs, UiO-66(Zr) and MIL-100(Fe), to evaluate further CO_2_ thermal chemisorption and reduction properties, although the other highly thermally stabilized MOFs are also candidates for catalytic reactions of CO_2_ reduction.

**Figure 6:**
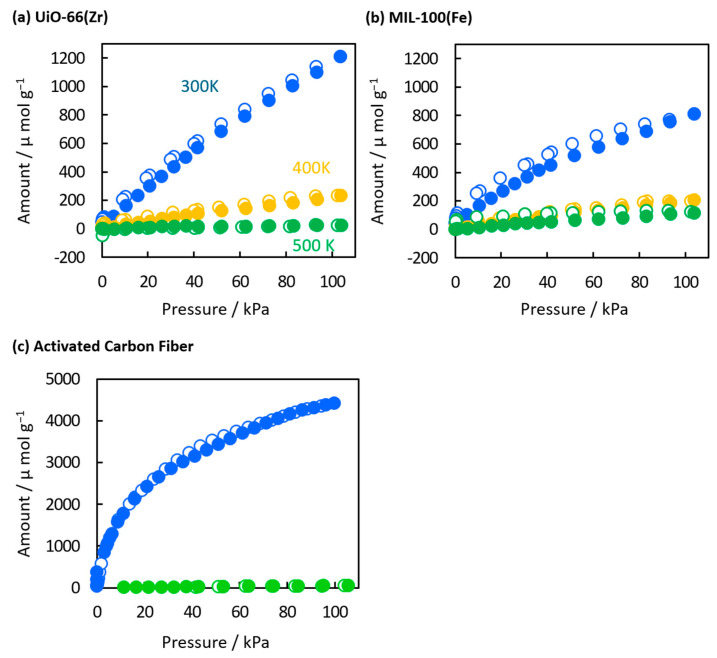
CO_2_ adsorption isotherms of (**a**) UiO-66(Zr), (**b**) MIL-100(Fe), and (**c**) activated carbon fiber at 300 K (blue circles), 400 K (orange circles), and 500 K (green circles). The closed and open symbols represent adsorption and desorption branches, respectively.

The temperature-dependent CO_2_ adsorption on MOFs was evaluated based on the adsorption isotherms of CO_2_ at 300, 400, and 500 K (Figure 6a,b). The CO_2_ adsorption capacities of UiO-66(Zr) and MIL-100(Fe) at 1 atm and 300 K were 1200 and 810 μmol g^−1^, respectively. The higher adsorption on UiO-66(Zr) can be attributed to its more substantial adsorption potential, i.e., smaller pore sizes and a more substantial adsorption site such as a bare Zr metal site. On the other hand, the adsorption capacity of MIL-100(Fe) at 500 K (115 μmol g^−1^) was higher than that of UiO-66(Zr) (21 μmol g^−1^). The maintenance of the adsorption amount on MIL-100(Fe) was especially anomalous because physical adsorption capacities are proportional to their adsorption potentials. This was obvious for an activated carbon fiber; the adsorption on activated carbon fiber drastically decreased from 4400 to 20 μmol g^−1^ by elevating the temperature from 300 K to 500 K (Figure 6c). Therefore, MIL-100(Fe) has more substantial adsorption sites than UiO-66(Zr) and activated carbon fiber. The high adsorption potential on MIL-100(Fe) has also been reported to be approximately 29 cm^3^ g^−1^ under low pressure at 298 K, 12.2 mmol g^−1^ under high pressure at 303 K [42,43], and 1.43 mmol g^−1^ at 1 bar and 298 K [44]. The strong adsorption potential of CO_2_ on MIL-100(Fe) primarily occurs at the Fe-metal sites, distinguishing MIL-100(Fe) from other MOF materials [43,45,46]. The slight hysteresis in the adsorption and desorption curves of MIL-100(Fe) suggests CO_2_ chemisorption on the Fe-metal sites at elevated temperatures.

**Figure 7:**
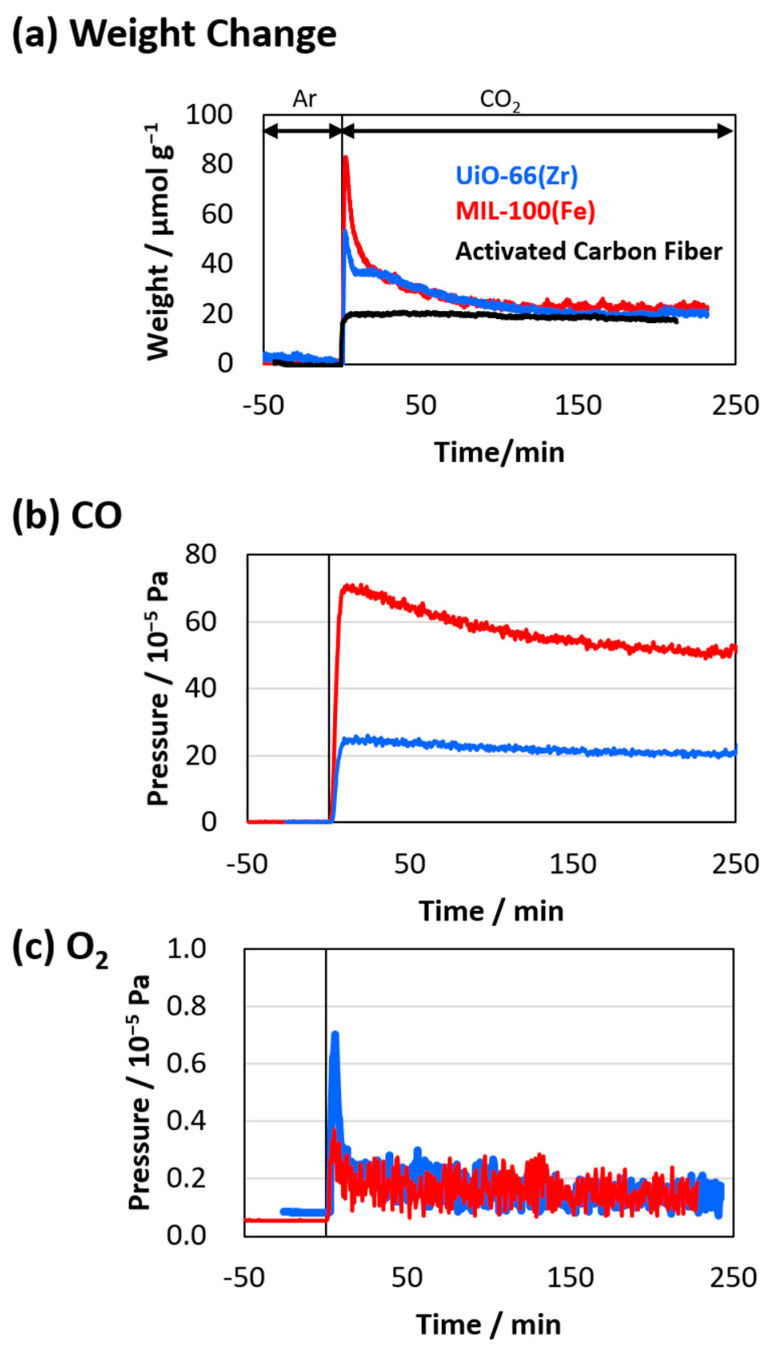
(**a**) CO_2_ adsorption on UiO-66(Zr) (blue curve) and MIL-100(Fe) (red curve) at 500 K. CO_2_ adsorption on activated carbon fiber is also shown as a reference (black curve). (**b**) CO and (**c**) O_2_ emissions are evaluated based on mass spectroscopies.

The CO_2_ adsorptions on UiO-66(Zr) and MIL-100(Fe) at 500 K were also investigated using TG analysis (Figure 7a) after their pretreatment under an Ar atmosphere for eight hours to eliminate contamination and guest molecules. The sample weights considerably increased after switching the gas flow from Ar to CO_2_, which can be attributed to the adsorption of CO_2_. The maximum CO_2_ amounts on UiO-66(Zr) and MIL-100(Fe) were 50 and 80 μmol g^−1^, respectively. A rapid adsorption was observed on the two MOFs, which can be attributed to the strong affinity of CO₂ molecules to the active sites (probably the metal sites) on the MOFs. However, the weights related to CO_2_ adsorption decreased to approximately 20 μmol g^−1^, which was rarely observed in common porous materials such as activated carbon fiber. In addition, the adsorption amounts were 20 and 110 μmol g^−1^ for UiO-66(Zr) and MIL-100(Fe), as shown in Figure 6. The amounts evaluated from the adsorption and TG measurements were similar for the UiO-66(Zr). In contrast, those were considerably different for the MIL-100(Fe). Therefore, we consider that any chemical reactions were involved in the CO_2_ adsorption process, especially for MIL-100(Fe). We assumed that the weight reductions were attributed to the decomposition of the adsorbed CO_2_ into CO and O_2_, which were hardly observed in the adsorption measurements, especially using a volumetric adsorption apparatus. CO and O_2_ emissions during the adsorption of the CO_2_ flow on UiO-66(Zr) and MIL-100(Fe) at 500 K were then examined using mass spectroscopy (Figure 7b,c). Considerable CO emissions were observed on MIL-100(Fe) compared to those on UiO-66(Zr), proving the thermo-catalytic reduction of CO_2_ on the MOFs. In addition, tiny amounts of O_2_ emissions were also observed. Thus, considerable CO_2_ decomposition into CO occurred, especially on MIL-100(Fe), via the thermo-catalytic reduction of CO_2_ on their surfaces. The CO_2_ reduction reaction rate was hardly estimated in the current work. However, the weight changes in Figure 7a suggested that the reduced amounts were 20–60 μmol g^−1^ in terms of the difference between the weight at the top peak and the equilibrium weight.

**Figure 8:**
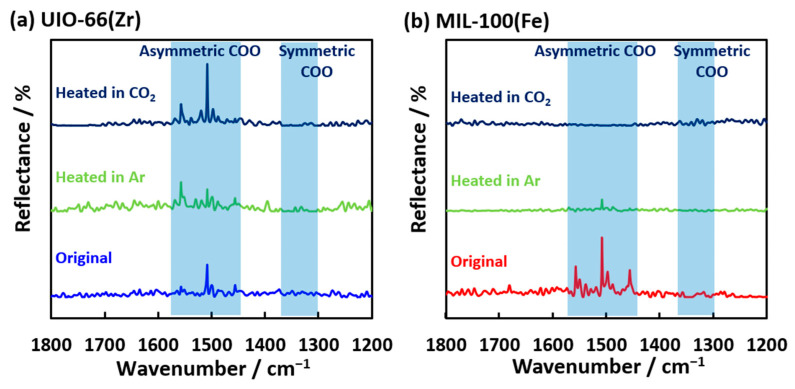
IR spectra of (**a**) UiO-66(Zr) and (**b**) MIL-100(Fe) before and after CO_2_ adsorption at 500 K.

**Figure 9:**
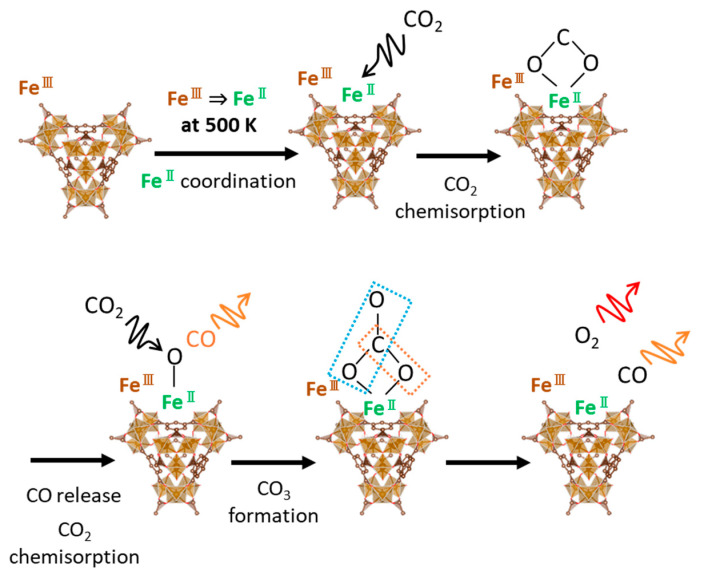
CO_2_ chemisorption and reduction mechanism on MIL-100(Fe) at 500 K.

The IR spectroscopies of CO_2_ and the related species were measured to analyze the chemisorbed and decomposed structures of CO_2_ adsorbed on the MOFs (Figure 8). Sharp reflectance bands were observed at 1460, 1500, and 1560 cm^−1^, which are attributed to asymmetric COO stretching for both UiO-66(Zr) and MIL-100(Fe) [47]. The reflectance band transformed into the other reflectance bands on UiO-66(Zr) when heated at 500 K under an Ar atmosphere. Moreover, the reflectance band of the asymmetric COO groups was recovered after the CO_2_ adsorption at 500 K, indicating the physical adsorption and/or chemisorption of CO_2_ on UiO-66(Zr). The reflectance bands of MIL-100(Fe) at 1600–1400 cm^−1^ were hardly observed after heating at 500 K. However, the broad band of the symmetric COO stretching at 1300–1400 cm^−1^ was weakly observed after the CO_2_ adsorption at 500 K compared to heating in the Ar atmosphere. These results suggest different forms of CO_2_ adsorption on the UiO-66(Zr) and MIL-100(Fe) surfaces. The electrochemical CO_2_ reduction on the two-dimensional MOF, PcNi-Co-O, had the intermediate state of *COOH, which was determined from the ATR-FTIR peak at 1400 cm^−1^ [48]. The CoO_4_ active sites worked as the substrate for the intermediate structure formation of CO_2_. The iron (II) site could be active in this work, as depicted in Figure 9. The CO_2_ adsorption and decomposition mechanism on MIL-100(Fe) was proposed and is shown in Figure 9, although the transition process of CO_2_ reduction was hardly observed. Fe(III) on MIL-100(Fe) is reduced to Fe(II) at 500 K [25,46,49]. Fe(II) moves to a bare site and interacts with CO_2_ gas. Next, the chemisorbed CO_2_ molecule is attached to Fe(II) in its symmetric OCO form and released as CO. The rest of the CO_2_ molecules are chemisorbed as carbonates on Fe(II), finally releasing CO and O_2_, confirmed by detecting CO and a slight amount of O_2_ in the mass spectra (Figure 7a,b).

## 4. Conclusions

The CO_2_ adsorption on UiO-66(Zr) and MIL-100(Fe) and their thermo-catalytic activities for CO_2_ reduction at 300–500 K were investigated. Both MOFs exhibit large specific surface areas owing to their nanoporous crystal structures, which were maintained after heating at 500 K and under a CO_2_ atmosphere. The CO₂ adsorption capacity of UiO-66(Zr) (1200 μmol g^−1^) was considerably higher than that of MIL-100(Fe) (810 μmol g^−1^) at 300 K despite their similar specific surface areas and pore volumes. However, the adsorption amount of MIL-100(Fe), 115 μmol g^−1^, was much larger than UiO-66(Zr) (21 μmol g^−1^) at 500 K. In addition, the CO_2_ adsorption on an activated carbon fiber at 500 K was drastically decreased to 0.4% of its original value at 300 K. The better performance of MOFs is due to their strong adsorption potential, resulting in unique CO_2_ adsorption properties, including CO_2_ chemisorption and reduction into CO, which was especially evident on MIL-100(Fe). The changes in the IR spectra corresponding to the carbonyl and OCO groups indicated the chemisorption, reduction, and release cycles of CO_2_ during the thermo-catalytic process on the bare Fe(II) sites of MIL-100(Fe). The success of MOFs as thermo-catalysts for CO_2_ reduction can be a basis for further advancing MOF thermo-catalysts for efficient and sustainable CO₂ capture strategies. Further studies on the quantitative analysis of CO_2_ reduction on MOFs should be conducted to develop MOF-based CO_2_ reduction catalysts.

## Figures and Tables

**Table 1:** Structural parameters of the two MOF samples.

Sample	S_BET_/m^2^ g^−1^	V_p_/cm^3^ g^−1^	d_p_/nm
UiO-66(Zr)	1020	0.50	2.1
MIL-100(Fe)	940	0.49	2.5

## Data Availability

The data that support the findings of this study are available from the corresponding author upon reasonable request.

## References

[1] Ghanbari T., Abnisa F., Wan Daud W.M.A. (2020). A review on production of metal organic frameworks (MOF) for CO_2_ adsorption. Sci. Total Environ..

[2] Diffenbaugh N.S., Burke M. (2019). Global warming has increased global economic inequality. Proc. Natl. Acad. Sci. USA.

[3] Notz R.J., Tönnies I., McCann N., Scheffknecht G., Hasse H. (2011). CO_2_ Capture for Fossil Fuel-Fired Power Plants. Chem. Eng. Technol..

[4] Yeh J.T., Resnik K.P., Rygle K., Pennline H.W. (2005). Semi-batch absorption and regeneration studies for CO_2_ capture by aqueous ammonia. Fuel Process. Technol..

[5] Chue K.T., Kim J.N., Yoo Y.J., Cho S.H., Yang R.T. (1995). Comparison of activated carbon and zeolite 13X for CO_2_ recovery from flue gas by pressure swing adsorption. Ind. Eng. Chem. Res..

[6] Wright P.A. (2008). Microporous Framework Solids.

[7] Xu R., Pang W., Yu J., Huo Q., Chen J. (2007). Chemistry of Zeolites and Related Porous Materials: Synthesis and Structure.

[8] Guo B., Chang L., Xie K. (2006). Adsorption of Carbon Dioxide on Activated Carbon. J. Nat. Gas Chem..

[9] Wahby A., Silvestre-Albero J., Sepúlveda-Escribano A., Rodriguez-Reinoso F. (2012). CO_2_ Adsorption on Carbon Molecular Sieves. Micropor. Mesopor. Mater..

[10] Liu Y., Wang Z., Zhou H.-C. (2012). Recent advances in carbon dioxide capture with metal-organic frameworks. Greenh. Gases Sci. Technol..

[11] Lawson H.D., Walton S.P., Chan C. (2021). Metal-Organic Frameworks for Drug Delivery: A Design Perspective. ACS Appl. Mater. Interfaces.

[12] Furukawa H., Cordova K.E., O’Keeffe M., Yaghi O.M. (2013). The chemistry and Applications of metal-organic frameworks. Science.

[13] Li J.-R., Ma Y., McCarthy M.C., Sculley J., Yu J., Jeong H.-K., Balbuena P.B., Zhou H.-C. (2011). Carbon dioxide capture-related gas adsorption and separation in metal-organic frameworks. Coord. Chem. Rev..

[14] Yu J., Balbuena P.B. (2013). Water Effects on Postcombustion CO_2_ Capture in Mg-MOF-74. J. Phys. Chem. C.

[15] Wang H.H., Hou L., Li Y.Z., Jiang C.Y., Wang Y.Y., Zhu Z. (2017). Porous MOF with Highly Efficient Selectivity and Chemical Conversion for CO_2_. ACS Appl. Mater. Interfaces.

[16] Emam H.E., Abdelhamid H.N., Abdelhameed R.M. (2018). Self-cleaned photoluminescent viscose fabric incorporated lanthanide-organic framework (Ln-MOF). Dyes Pigm..

[17] Fang Y., Yang Z., Li H., Liu X. (2020). MIL-100(Fe) and its derivatives: From synthesis to application for wastewater decontamination. Environ. Sci. Pollut. Res. Int..

[18] Quijia C.R., Lima C., Silva C., Alves R.C., Frem R., Chorilli M. (2021). Application of MIL-100(Fe) in drug delivery and biomedicine. Drug Deliv. Technol..

[19] Huang S., Yang K.-L., Liu X.-F., Pan H., Zhang H., Yang S. (2017). MIL-100(Fe)-catalyzed efficient conversion of hexoses to lactic acid. RSC Adv..

[20] Cmarik G.E., Kim M., Cohen S.M., Walton K.S. (2012). Tuning the Adsorption Properties of UiO-66 via Ligand Functionalization. Langmuir.

[21] Cao Y., Zhang H., Song F., Huang T., Ji J., Zhong Q., Chu W., Xu Q. (2018). UiO-66-NH_2_/GO Composite: Synthesis, Characterization and CO_2_ Adsorption Performance. Materials.

[22] Guesh K., Caiuby C.A.D., Mayoral Á., Díaz-García M., Díaz I., Sanchez-Sanchez M. (2017). Sustainable Preparation of MIL-100(Fe) and Its Photocatalytic Behavior in the Degradation of Methyl Orange in Water. Cryst. Growth Des..

[23] Katz M.J., Brown Z.J., Colón Y.J., Siu P.W., Scheidt K.A., Snurr R.Q., Hupp J.T., Farha O.K. (2013). A facile synthesis of UiO-66, UiO-67 and their derivatives. Chem. Commun..

[24] Cavka J.H., Jakobsen S., Olsbye U., Guillou N., Lamberti C., Bordiga S., Lillerud K.P. (2008). A New Zirconium Inorganic Building Brick Forming Metal Organic Frameworks with Exceptional Stability. J. Am. Chem. Soc..

[25] Horcajada P., Surblé S., Serre C., Hong D.-Y., Seo Y.-K., Chang J.-S., Grenèche J.-M., Margiolaki I., Férey G. (2007). Synthesis and catalytic properties of MIL-100(Fe), an iron(III) carboxylate with large pores. Chem. Commun..

[26] Dhakshinamoorthy A., Alvaro M., Hwang Y.K., Seo Y.-K., Corma A., Garcia H. (2011). Intracrystalline diffusion in Metal Organic Framework during heterogeneous catalysis: Influence of particle size on the activity of MIL-100 (Fe) for oxidation reactions. Dalton Trans..

[27] Ramsahye N.A., Trens P., Shepherd C., Gonzalez P., Trung T.K., Ragon F., Serre C. (2014). The effect of pore shape on hydrocarbon selectivity on UiO-66(Zr), HKUST-1 and MIL-125(Ti) metal organic frameworks: Insights from molecular simulations and chromatography. Micropor. Mesopor. Mater..

[28] Ragon F., Campo B., Yang Q., Martineau C., Wiersum A.D., Lago A., Guillerm V., Hemsley C., Eubank J.F., Vishnuvarthan M. (2015). Acid-functionalized UiO-66(Zr) MOFs and their evolution after intra-framework cross-linking: Structural features and sorption properties. J. Mater. Chem. A.

[29] Ladavos A.K., Katsoulidis A.P., Iosifidis A., Triantafyllidis K.S., Pinnavaia T.J., Pomonis P.J. (2012). The BET equation, the inflection points of N2 adsorption isotherms and the estimation of specific surface area of porous solids. Micropor. Mesopor. Mater..

[30] Asghar A., Iqbal N., Noor T. (2020). Ultrasonication treatment enhances MOF surface area and gas uptake capacity. Polyhedron.

[31] Yin D., Hu X., Cai M., Wang K., Peng H., Bai J., Xv Y., Fu T., Dong X., Ni J. (2022). Preparation, Characterization, and In Vitro Release of Curcumin-Loaded IRMOF-10 Nanoparticles and Investigation of Their Pro-Apoptotic Effects on Human Hepatoma HepG2 Cells. Molecules.

[32] Biswas S., Maes M., Dhakshinamoorthy A., Feyand M., De Vos D.E., Garcia H., Stock N. (2012). Fuelpurification, Lewis acid and aerobic oxidation catalysis performed by a microporous Co-BTT (BTT^3−^ = 1,3,5-benzenetristetrazolate) framework having coordinatively unsaturated sites. J. Mater. Chem..

[33] Ta H., Pham Ngoc T., Nguyen X.H., Son D. (2016). Metal—Organic Frameworks: State-of-the-art Material for Gas Capture and Storage. VNU J. Sci. Math. Phys..

[34] Yari Kalashgrani M., Babapoor A., Mousavi S.M., Feizpoor S., Hashemi S.A., Chiang W.-H., Lai C.w. (2023). Synthesis of Isoreticular Metal Organic Framework-3 (IRMOF-3) Porous Nanostructure and Its Effect on Naphthalene Adsorption: Optimized by Response Surface Methodology. Separations.

[35] Colombo V., Galli S., Choi H.J., Han G.D., Maspero A., Palmisano G., Masciocchi N., Long J.R. (2011). High thermal and chemical stability in pyrazolate-bridged metal–organic frameworks with exposed metal sites. Chem. Sci..

[36] Abid H.R., Azhar M.R., Iglauer S., Rada Z.H., Al-Yaseri A., Keshavarz A. (2024). Physicochemical Characterization of metal organic framework materials: A mini review. Heliyon.

[37] Abid H.R., Rada Z.H., Li Y., Mohammed H.A., Wang Y., Wang S., Arandiyan H., Tan X., Liu S. (2020). Boosting CO_2_ adsorption and selectivity in metal–organic frameworks of MIL-96(Al) via second metal Ca coordination. RSC Adv..

[38] Halder A., Lee S., Yang B., Pellin M., Vajda S., Li Z., Yang Y., Farha O., Hupp J. (2020). Structural reversibility of Cu doped NU-1000 MOFs under hydrogenation conditions. J. Chem. Phys..

[39] Nguyen P.T., Nguyen H.T., Pham H.Q., Kim J., Cordova K.E., Furukawa H. (2015). Synthesis and Selective CO_2_ Capture Properties of a Series of Hexatopic Linker-Based Metal-Organic Frameworks. Inorg. Chem..

[40] Ye X., Liu D. (2021). Metal–Organic Framework UiO-68 and Its Derivatives with Sufficiently Good Properties and Performance Show Promising Prospects in Potential Industrial Applications. Cryst. Growth Des..

[41] Saha D., Deng S. (2010). Structural Stability of Metal Organic Framework MOF-177. J. Phys. Chem. Lett..

[42] Mutyala S., Yakout S.M., Ibrahim S.S., Jonnalagadda M., Mitta H. (2019). Enhancement of CO_2_ capture and separation of CO_2_/N_2_ using post-synthetic modified MIL-100(Fe). New J. Chem..

[43] Oliveira L.T., Gonçalves R.V., Gonçalves D.V., de Azevedo D.C.S., Pereira de Lucena S.M. (2019). Superior Performance of Mesoporous MOF MIL-100 (Fe) Impregnated with Ionic Liquids for CO_2_ Adsorption. J. Chem. Eng. Data.

[44] Mei L., Jiang T., Zhou X., Li Y., Wang H., Li Z. (2017). A novel DOBDC-functionalized MIL-100(Fe) and its enhanced CO_2_ capacity and selectivity. Chem. Eng. J..

[45] Ahmed H.E., Albolkany M.K., El-Khouly M.E., El-Moneim A.A. (2024). Tailoring MIL-100(Fe)-derived catalyst for controlled carbon dioxide conversion and product selectivity. RSC Adv..

[46] Lv H., Zhao H., Cao T., Qian L., Wang Y., Zhao G. (2015). Efficient degradation of high concentration azo-dye wastewater by heterogeneous Fenton process with iron-based metal-organic framework. J. Mol. Catal. A Chem..

[47] Du J., Zhang F., Jiang L., Guo Z., Song H. (2023). Enhanced cobalt MOF electrocatalyst for oxygen evolution reaction via morphology regulation. Inorg. Chem. Commun..

[48] Zhang M.-D., Huang J.-R., Shi W., Liao P.-Q., Chen X.-M. (2023). Synergistic Effect in a Metal–Organic Framework Boosting the Electrochemical CO_2_ Overall Splitting. J. Am. Chem. Soc..

[49] Cheng R., Debroye E., Hofkens J., Roeffaers M.B.J. (2020). Efficient Photocatalytic CO_2_ Reduction with MIL-100(Fe)-CsPbBr_3_ Composites. Catalysts.

